# Rhythmic motor behavior explains individual differences in grammar skills in adults

**DOI:** 10.1038/s41598-024-53382-9

**Published:** 2024-02-14

**Authors:** Hyun-Woong Kim, Jessica Kovar, Jesper Singh Bajwa, Yasir Mian, Ayesha Ahmad, Marisol Mancilla Moreno, Theodore J. Price, Yune Sang Lee

**Affiliations:** 1 School of Behavioral and Brain Sciences, University of Texas at Dallas, Richardson, USA; 2 Callier Center for Communication Disorders, University of Texas at Dallas, Richardson, USA; 3 Department of Psychology, The University of Texas at Dallas, Richardson, USA; 4 Department of Neuroscience and Center for Advanced Pain Studies, University of Texas at Dallas, Richardson, USA; 5 Department of Speech, Language, and Hearing, The University of Texas at Dallas, Richardson, USA

**Keywords:** Psychology, Human behaviour

## Abstract

A growing body of literature has reported the relationship between music and language, particularly between individual differences in perceptual rhythm skill and grammar competency in children. Here, we investigated whether motoric aspects of rhythm processing—as measured by rhythmic finger tapping tasks—also explain the rhythm-grammar connection in 150 healthy young adults. We found that all expressive rhythm skills (spontaneous, synchronized, and continued tapping) along with rhythm discrimination skill significantly predicted receptive grammar skills on either auditory sentence comprehension or grammaticality well-formedness judgment (e.g., singular/plural, past/present), even after controlling for verbal working memory and music experience. Among these, synchronized tapping and rhythm discrimination explained unique variance of sentence comprehension and grammaticality judgment, respectively, indicating differential associations between different rhythm and grammar skills. Together, we demonstrate that even simple and repetitive motor behavior can account for seemingly high-order grammar skills in the adult population, suggesting that the sensorimotor system continue to support syntactic operations.

## Introduction

Rhythm is pervasive in a myriad of auditory events including music, language, and everyday listening sounds. Importantly, in the language domain, rhythmicity in continuous speech signals provides potent cues for tracking ongoing syntactic structures^1,2^. While previous theoretical frameworks situate language as a highly specialized faculty distinct from music^3,4^, a growing body of evidence demonstrates the relationship between musical rhythm and linguistic syntax^5–8^. For example, the ability to discriminate between pairs of short musical rhythms was positively associated with both receptive^6,8^ and expressive^5,7^ grammar proficiencies in typically developing children. Such rhythm-grammar connection may suggest co-optation of neurobiological resources shared by music and language^9^.

A core neuroanatomical substrate supporting rhythm processing is the sensorimotor system consisting of the basal ganglia, supplementary motor area, premotor cortex, and cerebellum. Indeed, synchronizing movements to musical rhythms is ubiquitous in human behaviors; listening to music often makes us spontaneously move to its rhythm. The sensorimotor brain regions are activated not only by rhythm production^10–12^, but also by passively listening to musical rhythms^13–15^. In particular, the basal ganglia and supplementary motor area have been shown to be sensitive to temporal regularity of musical rhythms that induces a sense of beat^16–19^. As such, it has been proposed that the motor circuitries connecting the basal ganglia to supplementary motor area support rhythm and beat perception by implicitly generating periodic actions towards upcoming beat timings^20^.

Given the crucial involvement of the sensorimotor system in rhythm perception, the motor component of rhythm processing may also explain individual differences in following rule-based temporal dynamics in the language domain, i.e., syntax. However, there are only a few studies that examined both beat synchronization and grammar skills in children^21,22^. In addition, although there are large individual differences in both motoric and perceptual rhythm skills in adults^23,24^ as well as in children^25^, whether such differences can be translated to grammar skills in the adult population remains elusive. In the present study, we addressed this issue by measuring multiple rhythm and grammar skills in a large sample of healthy young adults.

Sensorimotor rhythmic skill is often assessed via synchronized finger tapping to an isochronous (i.e., metronome) tone sequence; the consistency (or variability) of tapping is used to measure the sensorimotor synchronization performance^26^. In addition, endogenous rhythmic behavior has been evaluated by means of spontaneous motor tapping that involves finger tapping at one’s most natural and comfortable interval, often associated with ‘personally preferred’ or ‘optimal’ tempo^27,28^. Here we measured these motoric rhythm skills as well as a perceptual rhythm skill using a same/different rhythm discrimination task. For grammar measures, we employed two language tasks on spoken sentences wherein participants were asked to judge syntactic well-formedness of spoken sentences with either a subject- or object-relative center-embedded clause or to identify the grammatical agent linked to an action verb^29^,^30^. In addition, we assessed participants’ verbal working memory, which was used as covariates along with demographic information including age, gender, and music background. We predicted that performance on the rhythm tasks would be associated with performance on the grammar tasks, even after controlling for verbal working memory.

## Results

### Description of the behavioral tasks and outcomes

**Figure 1:**
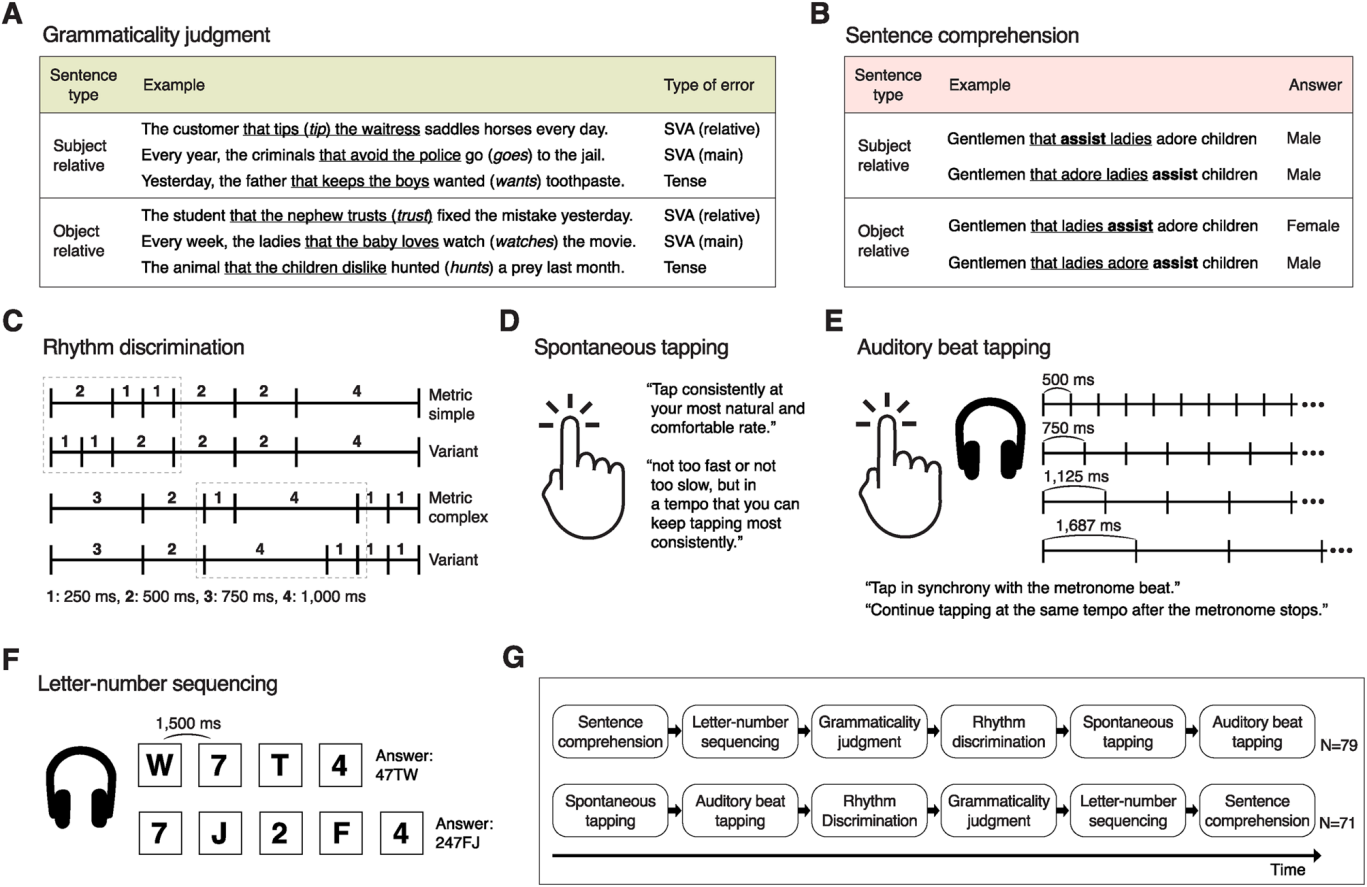
Overview of the experimental procedures. (**A**) Example sentences in grammaticality judgment task. Participants indicate if each spoken sentence is grammatically correct or not. Half of the sentences contain a subject-verb agreement (SVA) error or a past tense error. The relative clause is underlined, and the syntactic error is shown as italic in the parenthesis. (**B**) Example sentences in sentence comprehension task. Participants indicate the gender of individuals linked to an action verb, but not to four pre-designated preference verbs (love, adore, hate, and dislike), on each spoken sentence presented with or without a multi-talker babble noise. The relative clause is underlined, and the target action verb is in bold. (**C**) Schematic representation of rhythm sequences in rhythm discrimination task. Participants listen to each pair of rhythms and indicate if they were the same or different. (**D**) Spontaneous tapping task. Participants are instructed to tap consistently at their own tempo without external metronomes. (**E**) Auditory beat tapping task. Participants tap along with metronome beats presented in one of four tempos (inter-beat intervals of 500, 750, 1125, or 1687 ms) (synchronization phase) and continue tapping after the metronome stops (continuation phase). (**F**) Schematic representation of auditory sequence in letter-number sequencing task. Participants listen to a sequence of alternating letters and numbers and repeat them back in a sorted order. (**G**) Each participant completes the six behavioral tasks in one of the two orders shown. See Methods for more details.

**Figure 2:**
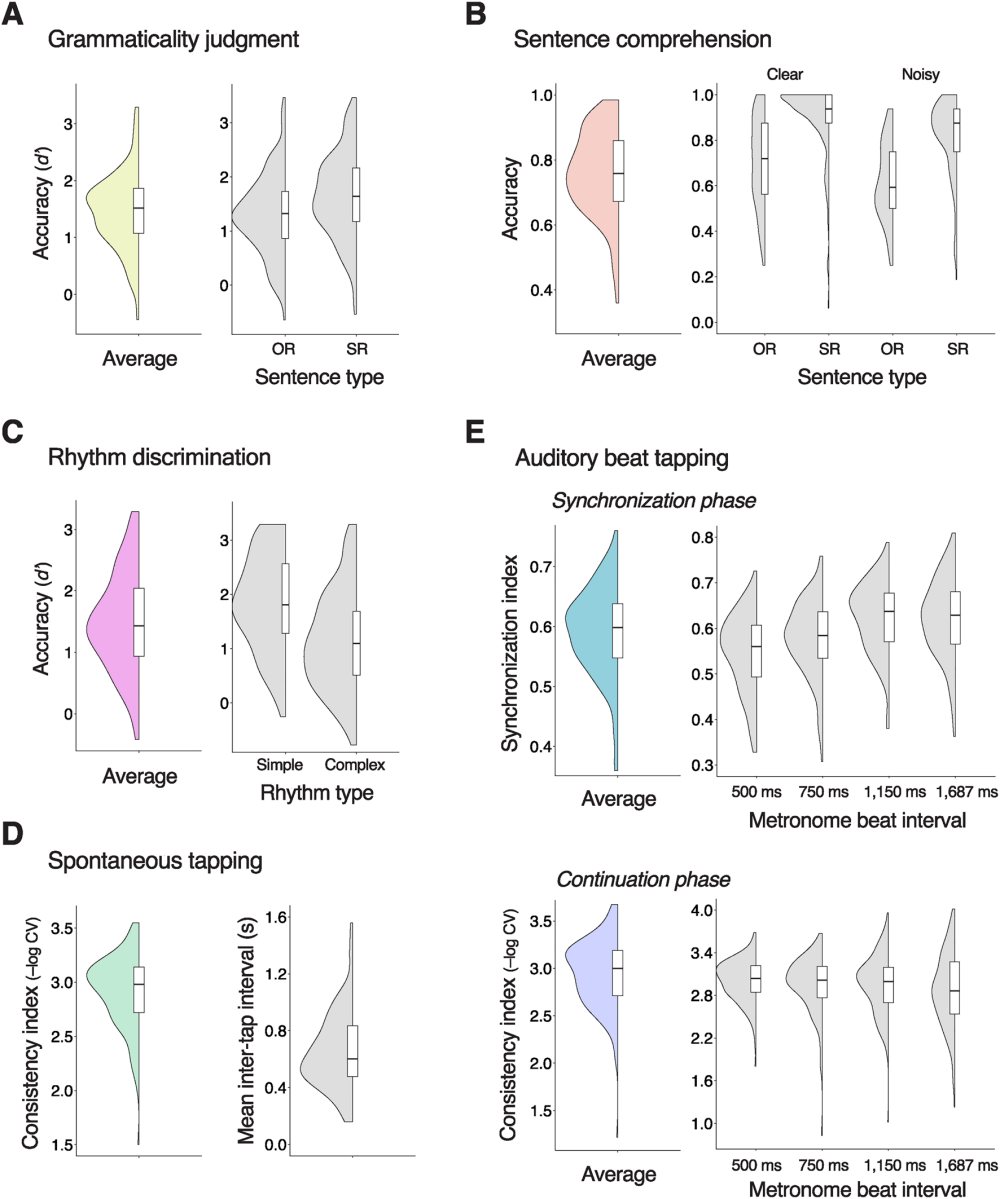
Rhythm and grammar performances. Half violin plots display the distributions of task performance. Box plots display the median (horizontal line within the box), the first quantile (lower boundary), and the third quantile (upper boundary). (**A**) Grammaticality judgment accuracy. (**B**) Sentence comprehension accuracy. (**C**) Rhythm discrimination accuracy. (**D**) Spontaneous tapping consistency (left) and mean tapping interval (right). (**E**) Auditory beat tapping consistency during synchronization phase (top) and continuation phase (bottom).

A total of 150 participants (mean age = 20.4 years, SD = 2.46) underwent two language tasks on spoken sentences (grammaticality judgment and sentence comprehension), three rhythm tasks (rhythm discrimination, spontaneous tapping, and auditory beat tapping), and a working memory task (letter-number sequencing) (Fig. 1). The behavioral results are summarized in Fig. 2 and Table 1.

**Table 1:** Mean raw scores (*M*) and standard deviations (*SD*) of demographic and behavioral variables.

Variable	*M*	*SD*	Median	Min	Max
Gender (F:M)	80:70	–	–	–	–
Age (years)	20.5	2.49	20	18	37
Years of musical training	4.24	4.69	3	0	17
Grammaticality judgment accuracy (*d*′)	1.47	0.66	1.52	− 0.44	3.67
Sentence comprehension accuracy (acc)	0.76	0.13	0.76	0.36	0.98
Rhythm discrimination accuracy (*d*′)	1.49	0.80	1.43	− 0.42	3.29
Spontaneous tapping consistency (− log CV)	2.91	0.33	2.98	1.5	3.55
Beat tapping consistency: synchronization (*SI*)	0.59	0.07	0.60	0.36	0.76
Beat tapping consistency: continuation (− log CV)	2.94	0.36	3	1.22	3.67
Letter-number sequencing accuracy (N correct)	12.8	3.44	12	4	24
Spontaneous tapping mean interval (s)	0.66	0.264	0.602	0.160	1.558

The grammaticality judgment task assessed participants’ ability to discern syntactic well-formedness on a series of spoken sentences that differed in syntactic complexity, containing either a subject-relative (SR) or object-relative (OR) center-embedded clause (Fig. 1A). Half of these sentences were grammatically correct while the other half had a morpho-syntactic error. As expected, participants performed worse on detecting grammatical errors in OR compared to SR sentences (Fig. 2A; *F*(1,149) = 38.1, *P* = 6 × 10^−9^), due to its non-canonical structure^31^.

The sentence comprehension task also consisted of a series of SR and OR sentences (all syntactically well-formed). Half of these sentences were mixed with multi-talker babble noise. In this task, participants identified the gender of a noun (e.g., boys) linked to an action verb (e.g., assist), while ignoring a noun related to any of the following preference verbs: love, adore, dislike, and hate (Fig. 1B). As was the case with the grammaticality judgment task, OR sentences yielded lower comprehension accuracy than SR sentences (*χ*^2^(1) = 527.8, *P* < 2 × 10^−16^). The background noise yielded lower accuracy (*χ*^*2*^(1) = 125.7, *P* < 2 × 10^−16^), especially for OR sentences, resulting in significant interaction with the sentence type (*χ*^*2*^(1) = 10.7, *P* = 0.001) (Fig. 2B).

In the rhythm discrimination task, participants determined whether pairs of short rhythmic sequences were the same or different (Fig. 1C). Half of the rhythms were metrically simple in that the inter-tone intervals were regularly arranged into groups of 1-s beat intervals, rendering a sense of beat^16^. The other half of rhythms were metrically complex, making it difficult to detect the underlying beat. Consistent with previous findings^16,32^, metrically complex rhythms were more difficult to discriminate than simple rhythms (Fig. 2C;* F*(1,149) = 105.6, *P* < 2 × 10^−16^).

In the spontaneous tapping task, participants were instructed to tap their right index finger consistently at their most natural and comfortable tempo (Fig. 1D). The average tempo (0.66 s) and its range (0.18–1.6 s) were similar to those in the previous report^28^. We gauged participants’ internal rhythm performance using a consistency index derived from the variance of inter-tap intervals (ITI; see Methods for more details).

In the auditory beat tapping task, participants tapped their right index finger to metronome beats with inter-beat intervals (IBIs) of 500, 750, 1125, and 1687 ms (synchronization phase) (Fig. 1E). These IBIs roughly correspond to 120, 80, 53, and 36 beats-per-minutes (BPM). Participants were also required to continue tapping consistently at the same tempo after the metronome stopped halfway through (continuation phase). A synchronization index was calculated from the uncertainty of the distribution of tap timings with respect to beat timings for the synchronization data, and a consistency index was computed from the ITI variance for the continuation data (see Methods for more details). We found that longer IBIs yielded more synchronized tapping responses during the synchronization phase (*χ*^*2*^(3) = 177.0, *P* < 2 × 10^−16^), while shorter IBIs yielded more consistent tapping during the continuation phase (*χ*^*2*^(3) = 16.4, *P* = 9 × 10^−4^) (Fig. 2E).

Lastly, we measured participants’ working memory using the letter-number sequencing task (Fig. 1F). In this task, participants were required to verbally re-organize a series of alternating numbers and letters in ascending orders (e.g., for K3F7R5, the correct answer is 357FKR). Working memory has been shown to be positively associated with a wide range of music and language abilities including sentence comprehension^33^ and grammaticality judgment^34^, as well as timing^35^, perceptual rhythm^25^, and motor rhythm^36^. Thus, we included the working memory measure as a covariate along with demographic variables of age, gender, and music background in the regression analyses.

### Multiple regression analysis

The regression results are listed in Table 2. The *P* values were adjusted with the false discovery rate (FDR) correction for multiple testing. We found that rhythm discrimination was significantly associated with grammaticality judgment (*b* = 0.24, *t* = 2.95, *P* = 0.009, FDR corrected), but not with sentence comprehension (*b* = 0.16, *t* = 1.93, *P* = 0.064, FDR corrected). By contrast, spontaneous tapping consistency was associated with both grammaticality judgment (*b* = 0.23, *t* = 2.73, *P* = 0.012, FDR corrected) and sentence comprehension (*b* = 0.26, *t* = 3.21, *P* = 0.006, FDR corrected). The strongest rhythm-grammar association among all pairwise regressions emerged between beat synchronization tapping and sentence comprehension (*b* = 0.40, *t* = 5.05, *P* = 1 × 10^−6^, FDR corrected), while this rhythm skill was not predictive of grammaticality judgment (*b* = 0.14, *t* = 1.63, *P* = 0.106, FDR corrected). Lastly, continuation tapping was related to both grammaticality judgment (*b* = 0.19, *t* = 2.11, *P* = 0.049, FDR corrected) and sentence comprehension (*b* = 0.26, *t* = 2.99, *P* = 0.009, FDR corrected).

**Table 2:** Standardized regression coefficients, *t* values, and uncorrected *P* values in predicting each of two grammar measures as a function of demographic variables and each of four rhythm measures.

Predictors	Grammaticality judgment			Sentence comprehension		
	*b*	*t*	*p*	*b*	*t*	*p*
Rhythm discrimination	**0**.**24**	**2**.**95**	**0**.**004**	0.16	1.93	0.056
*Verbal working memory*	*0*.*31*	*3*.*77*	< *0*.*001*	*0*.*30*	*3*.*57*	< *0*.*001*
*Years of musical training*	*0*.*11*	*1*.*52*	*0*.*131*	*0*.*18*	*2*.*44*	*0*.*016*
*Age*	*0*.*04*	*0*.*51*	*0*.*610*	− *0*.*01*	− *0*.*18*	*0*.*861*
*Gender (Male)*	− *0*.*01*	− *0*.*17*	*0*.*865*	*0*.*10*	*1*.*27*	*0*.*205*
Spontaneous tapping	**0**.**23**	**2**.**73**	**0**.**007**	**0**.**26**	**3**.**22**	**0**.**002**
*Verbal working memory*	*0*.*35*	*4*.*59*	< *0*.*001*	*0*.*30*	*3*.*96*	< *0*.*001*
*Years of musical training*	*0*.*07*	*0*.*82*	*0*.*411*	*0*.*11*	*1*.*37*	*0*.*172*
*Age*	*0*.*01*	*0*.*11*	*0*.*915*	− *0*.*05*	− *0*.*62*	*0*.*539*
*Gender (Male)*	− *0*.*04*	− *0*.*59*	*0*.*555*	*0*.*07*	*0*.*87*	*0*.*385*
Beat tapping: Synchronization	0.14	1.63	0.110	**0**.**40**	**5**.**05**	**< 0**.**001**
*Verbal working memory*	*0*.*38*	*4*.*80*	< *0*.*001*	*0*.*28*	*3*.*84*	< *0*.*001*
*Years of musical training*	*0*.*10*	*1*.*16*	*0*.*250*	*0*.*04*	*0*.*56*	*0*.*576*
*Age*	*0*.*02*	*0*.*30*	*0*.*764*	− *0*.*05*	− *0*.*65*	*0*.*516*
*Gender (Male)*	− *0*.*05*	− *0*.*62*	*0*.*539*	*0*.*02*	*0*.*27*	*0*.*790*
Beat tapping: Continuation	**0**.**19**	**2**.**11**	**0**.**037**	**0**.**26**	**2**.**99**	**0**.**003**
*Verbal working memory*	*0*.*35*	*4*.*31*	< *0*.*001*	*0*.*28*	*3*.*54*	< *0*.*001*
*Years of musical training*	*0*.*08*	*1*.*04*	*0*.*301*	*0*.*11*	*1*.*43*	*0*.*156*
*Age*	*0*.*02*	*0*.*21*	*0*.*836*	− *0*.*04*	− *0*.*55*	*0*.*585*
*Gender (Male)*	− *0*.*06*	− *0*.*79*	*0*.*434*	*0*.*04*	*0*.*48*	*0*.*630*

Significant values are in bold, and covariates are italicized.

To further learn whether different rhythm skills account for unique variance in grammar skills, we performed multiple regression analyses with all four rhythm measures—that were highly inter-related with each other—included as predictors. In this most stringent model, beat synchronization still robustly accounted for sentence comprehension (*b* = 0.40, *t* = 3.62, *P* = 4 × 10^−4^), whereas rhythm discrimination (*b* = 0.20, *t* = 2.22, *p* = 0.028) was predictive of grammaticality judgment, even after controlling for the other rhythm measures (Table 3). This suggests that there are dissociable aspects of temporal processing in the relationship between rhythm and grammar skills.

**Table 3:** Standardized regression coefficients, *t* values, and uncorrected *P* values in predicting each of two grammar measures as a function of demographic variables and all four rhythm measures.

Predictors	Grammaticality judgment			Sentence comprehension		
	*b*	*t*	*p*	*b*	*t*	*p*
Rhythm discrimination	**0**.**20**	**2**.**22**	**0**.**028**	0.02	0.21	0.834
Spontaneous tapping	0.19	1.68	0.095	0.11	0.98	0.327
Beat tapping: Synchronization	− 0.03	− 0.03	0.764	**0**.**40**	**3**.**62**	**< 0**.**001**
Beat tapping: Continuation	− 0.01	− 0.10	0.918	− 0.09	− 0.70	0.483
*Working memory*	*0*.*29*	*3*.*55*	< *0*.*001*	*0*.*28*	*3*.*51*	< *0*.*001*
*Years of musical training*	*0*.*07*	*0*.*79*	*0*.*429*	*0*.*04*	*0*.*46*	*0*.*648*
*Age*	*0*.*02*	*0*.*26*	*0*.*792*	− *0*.*05*	− *0*.*67*	*0*.*502*
*Gender (Male)*	− *0*.*02*	− *0*.*30*	*0*.*764*	*0*.*03*	*0*.*40*	*0*.*688*

Significant values are in bold, and covariates are italicized.

## Discussion

Although a growing body of research has demonstrated connections between musical rhythm and linguistic grammar skills^5–8^, the evidence has been limited in children and mostly to a perceptual rhythm skill, i.e., musical rhythm discrimination. In the present study with 150 healthy young adults, we demonstrated that a set of rhythm skills in both receptive and expressive domains were associated with receptive grammar tasks. Notably, even simple and repetitive motor behavior involving spontaneous or synchronized finger tapping was predictive of comprehension on spoken sentences that varied in syntactic structure. This finding extends the existing evidence beyond the perceptual rhythm toward expressive/motoric rhythm skills, as well as from children to young adults. Together, these results suggest that common neurobiological mechanisms may be at play in both rhythm and syntactic processing^9^, contributing to the association between individual differences in rhythm and grammar that persists into adulthood.

The present findings may shed light on the role of the motor system in auditory syntactic processing^37^, by showing that even a relatively simple motor task such as spontaneous or continued finger tapping explained both grammaticality judgment and syntactic interpretation on spoken sentences. Spontaneous rhythmic behavior has been theorized to reflect activity of self-sustaining internal oscillators^27,28^, which is likely regulated by cortico-striatal motor circuits^38,39^. Our data speak to the functional role of internal rhythmicity in readily analyzing syntactic structures during auditory sentence processing. In addition, beat synchronization tapping had a robust explanatory power in predicting sentence comprehension performance, which was significant even when controlling for the effects of the other rhythm measures. Given that sensorimotor synchronization to external rhythm is thought to rely on predictive timing mechanisms^26,40^, the current finding supports the idea that temporal prediction (e.g., when the next event occurs) may play a functional role in syntactic prediction (e.g., what word comes next given the preceding words in a sentence)^37,41^.

By contrast, the synchronized tapping measurements had a less robust relationship with grammaticality judgement performance, suggesting that this task may require some different mechanisms that cannot be solely explained by (forward) temporal prediction, such as (backward) re-analysis of preceding syntactic contents^42,43^. The re-analysis of temporal structure may have been captured by the significant relationship between rhythm discrimination and grammaticality judgment. This is perhaps because both tasks require back-and-forth comparison of words or tones in relation to the preceding ones to achieve timely judgement of the linguistic or rhythmic structure. This finding is in line with the idea that musical rhythm processing may recruit neurobiological resources for rule-based temporal processing shared by syntax processing system^9^.

Together, the current results suggest that different rhythm skills may uniquely contribute to accounting for different grammar skills. This underscores the importance of assessing multiple rhythm skills to gain a more complete picture of the rhythm-grammar relationship, which may be manifested through multiple neurocognitive mechanisms such as precise temporal predictions and re-analysis of temporal structures. Moreover, from a broader perspective, our findings suggest that the relationship between music and language may not be a unitary construct, but rather a consequence of multiple mechanisms shared by different aspects of music and language. For instance, phonological processing may be correlated more dominantly with components of rhythm processing that tap into precise auditory encoding^44,45^. Thus, future investigation of a wide range of music and language tasks using a well-powered sample may provide a more comprehensive understanding of the multifaceted associations between music and language.

In sum, the current study provides evidence for the association between expressive rhythm skills and receptive grammar skills in an adult population. This is in line with clinical observations that children with developmental language disorder often exhibit deficits in producing rhythmic movements^46^, which might build upon common genetic underpinnings^47,48^. Moreover, we found different rhythm skills uniquely explained different grammar skills, suggesting that there are dissociable aspects of temporal processing in the rhythm-grammar relationship. Our findings suggest that what has been regarded as a core linguistic operation, i.e., syntax, is associated with domain-general temporal processing in the sensorimotor system.

## Methods

### Participants

150 participants were recruited from the University of Texas at Dallas (80 females, 18–37 years, mean age = 20.4 years, *SD* = 2.5 years). An a priori power analysis was performed using G × Power version 3.1, with an alpha level of 0.05 × 8 = 0.0063, considering that there were eight pairwise multiple regression tests to be performed (see below). The result showed that at least 118 participants were required to find a medium effect size (f^2^ = 0.15, α error = 0.0063, Power = 0.95) with five predictors in a multiple regression analysis.

All participants spoke English as a primary language, had normal vision and hearing, and had no self-reported history of cognitive, developmental, or neurological disorders. All participants provided written informed consent to participate in the study. Participants’ musical experience was quantified as the number of years of actively practicing one or multiple musical instruments including voice (Table 1). The experimental protocols were approved by the University of Texas at Dallas Institutional Review Board (IRB-21-109) and conducted in accordance with the Declaration of Helsinki. All methods were carried out in agreement with the relevant guidelines and regulations.

### Experimental procedures

The experiment consisted of the following behavioral tasks: two for grammar (grammaticality judgment, sentence comprehension); three for rhythm (rhythm discrimination, spontaneous tapping, auditory beat tapping); one for verbal working memory (letter-number sequencing) (Figs. 1, 2). Each participant completed the tasks in either of two fixed orders, as shown in Fig. 1G. The experimental procedures were conducted using Matlab R2021 (Mathworks, MA) in a dimly lit sound-proof booth. All auditory stimuli were presented at a preset volume (70 dB SPL) through Sennheiser HD-280 headphones. We used Google Text-to-Speech to generate spoken sentence stimuli for the grammar tasks and verbal items for the working memory task. The speaker voice was set to an American-English speaking male for the language tasks and a female for the working memory task.

#### Grammaticality judgment task

The language materials consisted of 48 sentences, each of which contained either a subject-relative (SR) or object-relative (OR) center-embedded clause and a time adverb phrase (e.g., every week, last year) (Fig. 1A). Half of the sentences contained a morpho-syntactic error of one of three types: a subject-verb agreement error within the relative phrase, a subject-verb agreement error in the main phrase, or a tense error (Fig. 1A). The sentence type (SR and OR) and the type of error were counterbalanced across trials. In each trial, participants listened to a spoken sentence and judged whether the sentence was grammatically correct or not via button press (right arrow for ‘grammatical’ and left arrow for ‘ungrammatical’). Participants completed 8 practice trials, using sentences reserved only for practice (these sentences did not appear in the main task), with no visual presentation of the sentences. They received visual feedback after each response (‘Correct’ or ‘Incorrect’) and were able to immediately repeat the practice trial if they chose. There was no time constraint in this task. Participants underwent 48 trials presented in a randomized order without feedback during the main task. A 15-s break was provided every 12 trials. We computed a d-prime (*d*′) score for each sentence type, which was obtained by calculating the difference of the z-scores between hit and false alarm rates. The rates of 0 and 1 were adjusted to prevent an indefinite *d*′ score^49^.

#### Sentence comprehension task

The language materials were comprised of 64 base sentences, each consisting of six words: a male noun (e.g., men, sons, kings, etc.), a female noun (e.g., women, daughters, queens, etc.), a gender-neutral noun (e.g., children, farmers, artists, etc.), a relative pronoun ‘that’, a transitive action verb (e.g., help, protect, tease, bully, etc.), and one of four transitive preference verbs: love, adore, hate, and dislike. The six words were arranged to contain either an SR or OR center-embedded structure by switching the position of the noun and the verb within the relative clause (Fig. 1B). An action verb and a preference verb were located in either the main or relative clause. Half of the sentences were mixed with a background noise of multi-talker babble consisting of two male and two female speakers at a signal-to-noise ratio of − 1 dB. The sentence type (i.e., SR or OR), the clarity of the sentence (i.e., clear or noisy), the action verb location (i.e., main or relative clause), and the gender of the agent (i.e., female or male) were fully counterbalanced across trials. In each trial, participants listened to a spoken sentence and indicated the gender of the individuals performing an action, while disregarding those who love/adore/hate/dislike others, by pressing either the ‘male’ (left arrow) or ‘female’ (right arrow) key within 3 s. After the task instruction, participants underwent 8 practice trials during which the corresponding sentences were concurrently presented on the screen. They were provided with visual feedback and (if needed) additional verbal instruction from the experimenter following each response. Then they received 16 more trials with visual feedback but without visual presentation of the sentences. During the main task, participants completed a total of 64 trials presented in a randomized order without feedback and without visual presentation of the sentences. The sentences used during the practice trials were not presented. There was a 15-s break after every 16 trials.

Percent accuracy for each combination of sentence type and clarity conditions is displayed in Fig. 2B. We used a mixed effects logistic regression model (glmer in the *lme4* package)^50^ to analyze the binary correct/incorrect responses, with the factors of sentence type (SR and OR), clarity (clear and noisy), and an interaction between the two as fixed effects and with a random intercept of participant. We evaluated statistical significance using the Type III Wald chi-square tests in the *car* package^51^.

#### Rhythm discrimination task

Rhythm stimuli were comprised of 10 metrically simple and 10 metrically complex rhythmic tone sequences and their respective variants, chosen from a set of rhythms used in Grahn and Brett^16^. Each rhythm consisted of seven or eight woodblock sounds (20 ms in duration) with intervals of 250, 500, 750, or 1000 ms between the sounds, spanning 3 s. A variant of each rhythm was made by switching the order of two adjacent intervals in the rhythm (Fig. 1C). On each trial, participants listened to a pair of rhythms presented sequentially with a 2-s interval and judged whether the two rhythms were the same or different by pressing either the ‘same’ (left arrow) or ‘different’ (right arrow) key. There was no time constraint in this task. Each rhythm was presented twice, once paired with itself (i.e., ‘same’) and once paired with its variant (i.e., ‘different’), resulting in a total of 40 trials presented in a randomized order. Participants performed two practice trials (one ‘same’ and one ‘different’) with feedback using one of the 10 simple rhythms and its variant. Participants were allowed to replay the practice if they chose and hear the same two rhythm pairs again. No feedback was given during the main task. We computed a *d*′ score for each rhythm type. The *d*′ data were analyzed using a repeated-measures ANOVA with a within-subject factor of rhythm type (metric simple and metric complex).

#### Spontaneous tapping task

In the spontaneous tapping task, participants were instructed to tap the space bar with their right index finger consistently at their most natural and comfortable rate. They were encouraged not to tap too fast or too slow but at a tempo that they could keep tapping most consistently (Fig. 1D). Each of the four trials was terminated after 43 taps on the space bar (i.e., 42 ITIs). There was a short practice with 15 taps. The task was performed prior to the auditory beat tapping task (Fig. 1G), to prevent influence of exposure to metronome beats on spontaneous motor tempo. The first two ITIs were discarded form each trial. We obtained a mean ITI from the remaining 40 ITIs, which was calculated after excluding ITIs exceeding the initial mean ITI ± 3 standard deviations. We obtained a consistency index from a coefficient of variation (CV) of ITIs, i.e., a standard deviation of the remaining ITIs divided by the mean ITI. The consistency index was computed as − log (CV) for each trial and averaged across the four trials (Fig. 2D).

#### Auditory beat tapping task

Each trial of the task started with synchronization and ended with continuation. During the former phase, participants tapped the space bar with their right index finger in synchrony with a metronome beat (i.e., isochronous woodblock sounds each with 20 ms in duration). They were instructed to start tapping after the first four beats, accompanied by a countdown timer on the screen. The metronome stopped immediately after 21 taps (i.e., 20 ITIs), after which the continuation phase began wherein participants continued tapping consistently while trying to maintain the same tempo without metronome beats. The continuation phase lasted until receiving 21 tapping responses. Each trial used one of four IBIs, equally spaced in log scale: 500, 750, 1125, and 1687 ms (Fig. 1E). Each of these four tempos was repeated four times for a total of 16 trials. The presentation order of the four tempos was pseudo-randomized within each four-trial set, such that there was no transition from shortest to longest IBI or vice versa. Participants underwent a practice trial with an IBI of 850 ms and were allowed to repeat the practice until understanding the instruction. A 30-s break was given after each set.

We analyzed beat tapping data during the synchronization and continuation phases separately. For the synchronization tapping data, we computed a circular metric (*SI*) based on Shannon entropy (*SE*):

$$SE = - \mathop \sum \limits_{i = 1}^{M} p\left( i \right)\ln p\left( i \right), \quad SI = 1 - \frac{SE}{{\ln N}}$$

where *M* is the number of bins, covering the range from − 180° to + 180°; *p*(*i*) is the probability of tap timings (i.e., relative phase angles with respect to nearest beat timings) assigned to *i*_th_ bin; *N* is the total number of tapping responses. The bin size was set to 15° based on an optimal bin search procedure described in Kim et al.^25^ The *SE* quantifies the degree of spreading of a data distribution, i.e., the relative phase distribution in our data. We derived a synchronization index (*SI*) from *SE* as shown in the formula above. *SI* ranges from 0 (all responses occurred in different bins) to 1 (all responses occurred in a single bin), exhibiting a less skewed distribution compared to other consistency measures (e.g.,^23^). For the continuation tapping data with no reference beat timings, we computed a consistency index in the same way as for the spontaneous tapping data. The synchronization and consistency indices were computed for each tapping trial and averaged across the four trials for each tempo condition. The synchronization and consistency indices were analyzed using the Friedman test, a nonparametric test for repeated-measures data, with a within-subject factor of IBI (500, 750, 1125, and 1687 ms).

#### Letter-number sequencing task

The verbal items consisted of a set of spoken letters (‘C’, ‘D’, ‘F’, ‘H’, ‘J’, ‘K’, ‘L’, ‘P’, ‘Q’, ‘R’, ‘S’, ‘T’, ‘W’) and numbers (from ‘one’ to ‘nine’). On each trial, participants heard a list of alternating numbers and letters presented via headphones with a 1.5-s interval (Fig. 1F). After presentation of the sequence, they were required to say the numbers in order from the smallest to the largest and then the letters in alphabetical order. An experimenter manually recorded the correctness. There were two practice trials with a list length of three and four. The main task started from a set of four trials with three list items each, and the list length increased after every four trials until participants failed to recall all trials in each length, after which the experiment ended. The largest length was eight, resulting in a maximum of 24 trials. The letter-number sequencing score was computed as the number of trials correctly recalled.

### Multiple linear regression analysis

For each of the behavioral tasks, we used a representative outcome by collapsing scores across task conditions (Fig. 2). For example, a single measure was computed for synchronization by averaging consistency measures (*SI*) across four IBI conditions. A multiple linear regression analysis was performed for each combination of the two grammar and four rhythm measures, in which one of the four rhythm measures, working memory (i.e., letter-number sequencing score), gender, age, and years of musical training were entered as independent variables into the model to predict each of the two grammar measures (Table 2). The subsequent *P* values of the regression coefficients were adjusted with the false discovery rate (FDR) procedure (α = 0.05) to correct for multiple testing.

## Data Availability

The datasets generated and analyzed during the current study are available from the corresponding author on reasonable request.
